# Ageing and autism: A longitudinal follow-up study of mental health and quality of life in autistic adults

**DOI:** 10.3389/fpsyg.2022.741213

**Published:** 2022-08-23

**Authors:** Amanda Roestorf, Patricia Howlin, Dermot M. Bowler

**Affiliations:** ^1^Autism Research Group (ARG), Department of Psychology, City, University of London, London, United Kingdom; ^2^Department of Clinical Psychology, Institute of Psychiatry, Psychology and Neuroscience, King’s College London, London, United Kingdom

**Keywords:** autism, ageing, mental health, quality of life, follow-up studies

## Abstract

**Background:**

Poor mental health is known to adversely affect functional abilities, social isolation, and quality of life (QoL). It is, therefore, crucial to consider the long-term impacts of mental health conditions as autistic adults grow older.

**Objectives:**

To explore, in a group of community-based autistic adults, the extent of: (i) autistic traits, co-occurring physical and mental health conditions; (ii) age-related differences in those conditions, and changes over time; and (iii) their impact on everyday living and QoL.

**Method:**

About Sixty-eight autistic adults (aged 19–80 years) participated in the first study (T1); 49 participants from T1 took part in a follow-up at T2 (mean retest interval 2.4 years). Standardised self-report measures of autistic traits, mental health, and QoL were completed at both time points.

**Results:**

Over two-thirds (71%) of autistic adult participants experienced at least one co-occurring condition, and over a third (37%) met the criteria for three or more co-occurring conditions. Mental and physical health difficulties were related to autistic traits and difficulties in everyday life and were consistent predictors of poor QoL at T1 and T2.

**Conclusion:**

Mental health difficulties in autism persisted into older age and did not improve over time. These findings have important implications for mental health provision for autistic adults in older age.

## Introduction

There is an increasing drive for support of older adults in the general population, related to cognitive change, well-being, social isolation, and physical healthcare (e.g., Wright et al., 2016; Kelly et al., 2017; Wu, 2020). Studies in gerontology provide insights into the selective challenges of ageing, and the strategies that enable older adults to maintain cognitive functions (e.g., Salthouse, 2004), social integration, and better quality of life (QoL; Hornby-Turner et al., 2017). There are various definitions of QoL and Subjective well-being (SWB) but both concepts encompass domains of physical, psychological, environmental, and social well-being. The World Health Organization (2002), in their report on *Active Ageing* suggests that 60 years of age should be a marker of “older” adulthood, but caution that:

*“chronological age is not a precise marker for the changes that accompany ageing… [since] There are dramatic variations in health status, participation and levels of independence among older people of the same age”*

Autism spectrum disorder (henceforth “autism”^1^) is a lifelong neurodevelopmental condition that is estimated to occur in at least 1% of the general population (American Psychiatric Association, 2013; Lord et al., 2020). Autism is characterized by a specific yet diverse profile of characteristics that include differences in social communication and social interactions; a strong need for routine and sameness that includes differences in information processing and thinking, specific patterns of interests, and sensory sensitivities (Lord et al., 2018). In turn, these differences can affect everyday functioning and autonomy, social relationships, mental health, and QoL (Shattuck et al., 2011; Geurts and Vissers, 2012; Howlin et al., 2013; Fortuna et al., 2015; Lever and Geurts, 2016). In addition, recent evidence suggests that increased difficulties related to depression, sleep quality, and general psychological well-being are also determinants of poor QoL (Lawson et al., 2020).

There has been relatively little systematic research into the impact of ageing among autistic adults, whether diagnosed in childhood or later life (Mason et al., 2022; and see Magiati et al., 2014; Steinhausen et al., 2016, for systematic reviews). Information on the prevalence or persistence of mental health difficulties in older autistic adults is particularly limited; similarly, little is known about QoL changes in older age or the degree to which autism-related difficulties and mental health affect QoL (e.g., Howlin and Taylor, 2015; Roestorf and Bowler, 2016; Wise et al., 2017; Mason et al., 2018). However, there is evidence that autistic adults experience more physical and mental health difficulties than age-matched non-autistic peers (Croen et al., 2015; Hirvikoski et al., 2016; Zerbo et al., 2019; but see Lever and Geurts, 2016), with approximately 70–80% of autistic individuals having co-occurring physical and/or mental health conditions (e.g., Bishop-Fitzpatrick and Rubenstein, 2019; Hand et al., 2020). These difficulties are exacerbated by the lack of access to appropriate services in adulthood and across the lifespan (Parr, 2016; Wright et al., 2016; Robison, 2019). Some recent research suggests a generally poorer QoL in older autistic adults (e.g., Van Heijst and Geurts, 2015; Bishop-Fitzpatrick et al., 2016; Roestorf and Bowler, 2016; Mason et al., 2018; Yarar Zivrali et al., in press; and see Ayres et al., 2017 for a meta-analysis) although findings are inconsistent (see Chiang and Wineman, 2014 for a review). Factors related to social support, long-term relationships, engaging in meaningful employment, and lifestyle autonomy have been linked to positive mental health and improved QoL (e.g., Ratto and Mesibov, 2015; Van Heijst and Geurts, 2015; Lever and Geurts, 2016; Mason et al., 2018; Park et al., 2019). However, the cumulative effects of long-term, co-occurring, physical or psychiatric conditions on everyday functioning and QoL are largely unknown (Howlin and Moss, 2012; Howlin et al., 2013; Kats et al., 2013). Similarly, little is known about the availability or effectiveness of health, care and social support services to accommodate the individual differences of older autistic adults who may need continued support related to autistic traits, mental/physical health difficulties, or daily living skills (Mason et al., 2019; Charlton et al., 2021, 2022; Lord et al., 2021; Oakley et al., 2021). Thus, more longitudinal research is needed to evaluate the effects of long-term co-occurring conditions and their relation to QoL in older age (Michael, 2016; Oakley et al., 2021). Stress and anxiety-related difficulties have substantial implications for the social functioning, cognitive abilities, and adaptive behaviours of autistic individuals (Maisel et al., 2016; Wallace et al., 2016; South et al., 2017), and are further compounded by intolerance of uncertainty, and aversiveness to emotional experiences whilst simultaneously experiencing difficulties identifying and interpreting emotions (e.g., Maisel et al., 2016). Because of the complex associations between the clinical features of anxiety and autism, it remains important for clinicians to dissociate core autistic symptoms from mental health conditions and physical health, when considering the primary care and service needs of autistic people across the lifespan (Roestorf et al., 2019; Oakley et al., 2021). A critical evaluation is needed of individualised long-term support needs, alongside physical and mental health as an autistic person grows older (e.g., Charlton et al., 2022).

The present study focuses primarily on depression and anxiety symptoms since these are the most common mental health conditions in autism (e.g., Maisel et al., 2016; Hollocks et al., 2019). The negative effects of depression are far-reaching in terms of cognitive, social and psychological functioning, reduced QoL, increased disability, and premature mortality (McClintock et al., 2010; Khanna et al., 2014; Mason et al., 2019; Lawson et al., 2020), and these effects may be exacerbated in individuals with co-occurring intellectual disability and/or neurodevelopmental conditions including autism (e.g., Coppus, 2013; Ratto and Mesibov, 2015). Long-term mental health difficulties, such as depression, are associated with increased risk of neurocognitive disorders (i.e., dementia; Bauman, 2010), although whether this is increased for autistic individuals is unknown (e.g., Hategan et al., 2017).

### Background to the present study

As part of a larger programme of work on ageing in autism, participants completed a wide range of assessments including measures of autism symptomatology, social and behavioural adaptive skills, mental health, quality of life, cognitive, language and memory, and a range of executive functions (see Roestorf and Bowler, 2016; Roestorf, 2018 and section “Measures” below). One part of the programme focused on age-related comparisons between autistic and non-autistic adults (see Roestorf and Bowler, 2016 and Roestorf, 2018); in the present paper, we describe findings from (i) a cross sectional comparison of younger and older autistic adults aged 19–80 years and (ii) a short-term longitudinal study of change over time. Our main aim was to identify any factors that might be related to adverse mental health (Schwartz and Meyer, 2010) and reduced quality of life of autistic people (McConachie et al., 2018; Mason et al., 2019; Lawson et al., 2020).

### Study aims

#### Study 1 [First time-point (T1); cross-sectional]

At T1, the study set out to explore (i) the extent of autistic traits and co-occurring physical and mental health difficulties in autistic adults; (ii) age-related *differences* in these areas through comparisons between younger and older autistic adults; and (iii) how these factors are associated with daily living and QoL.

#### Study 2 [Second time-point (T2); follow-up]

At T2, we followed-up participants from the T1 study. In addition to replicating the aims of Study 1, the principal aim at T2 was (i) to evaluate the profile of potential age-related *changes* over time in younger and older autistic adults, related to autistic traits, co-occurring mental health conditions, and QoL. Regression analyses explored (ii) how these factors were associated with QoL over time.

## Materials and methods

### Ethics

Ethical approval was obtained from the City, University of London Psychology Department Research Ethics Committee PSYETH (UPTD) 13/14 28, for the research project titled: *Age-Related Effects on Cognition and Quality of Life in Adults with Autism Spectrum Disorder*, published as part of the programme of work submitted for the first author’s PhD Thesis (Roestorf, 2018).

### Participants

#### Procedure for participant selection and recruitment

The study was advertised *via* the United Kingdom National Autistic Society website and online *via* the Twitter network of the first author. We aimed to recruit older adults and autistic women as these groups are greatly underrepresented in most autism research. Participants from the research databases at the Autism Research Group and online research recruitment portal at City, University of London were also invited to take part in the study. Informed consent was obtained from all participants prior to enrolment in the study and they were informed of their right to withdraw from the study at any time, without being disadvantaged. Participants were offered £25 as a gratuity for taking part in the research at each time point and received full reimbursement of travel expenses.

The relative lack of studies on ageing in autism means there is no consensus on the definition of ‘older’ age in this group. For the purpose of the present study, we included autistic adults across a wide age range, with ‘older’ adults being defined as those aged ≥50 years and ‘younger’ adults as those aged 18–49 years. Participants were assigned to ‘younger’ and ‘older’ age groups at T1 and remained assigned to those same groups at T2 follow-up.

To ensure that participants were able fully to understand all the task requirements English language proficiency was assessed using the Comprehensive Receptive and Expressive Vocabulary Test–Third Edition (CREVT-3; Wallace and Hammill, 2013). Two participants were excluded at this stage as they did not meet the standardised assessment criteria for English fluency (CREVT-3 overall language score > 70; population mean 100, SD 15; see Appendix 1 in Supplementary material).

#### Sample characteristics

Participants at T1 comprised 68 autistic adults aged 19–80 years (mean 44.1 years, SD 15.5 years), including 37 younger (mean 31.9 years, 10 female) and 31 older adults (mean 58.6 years; seven female). All participants had a formal diagnosis of autism, confirmed by a copy of clinical diagnostic reports obtained at enrolment. Age groups were matched on gender ratio (reported as male and female in this study, no participants identified as transgender or non-binary), years of formal education and general intellectual ability (IQ; measured by Wechsler Adult Intelligence Scales–Fourth Edition; WAIS-IV; Wechsler, 2008; see Table 1 for data, and see Appendix 1 in Supplementary material). Following the T1 study, participants were asked if they would be willing to take part in a subsequent follow-up study (T2).

**Table 1:** T1 characteristics of younger and older adults.

Measure	Age group (*N* = 68)		Statistics		
	Younger (*n* = 37) Mean (SD)	Older (*n* = 31) Mean (SD)	*t(66)*	*p*	*Cohen’s d*
Age (yrs)	31.89 (8.02)	58.61 (7.36)	**−14.07**	**<0.001**	**−3.43**
YFE^a^	14.80 (2.38)	14.36 (2.90)	<1.00	0.69	0.17
FSIQ^b^	108.89 (14.91)	112.19 (18.95)	<−1.00	0.07	−0.19
VCI^b^	111.14 (15.71)	115.87 (16.49)	−1.21	0.36	−0.29
PRI^b^	110.49 (16.79)	111.16 (14.18)	<−1.00	0.17	−0.04
WMI^b^	87.50 (14.10)	91.92 (22.66)	<−1.00	0.07	−0.23
PSI^b^	94.68 (19.51)	101.27 (16.86)	−1.10	0.22	−0.31

a YFE, Years of Formal Education.

b FSIQ, Full-Scale IQ; Index Scores: VCI, Verbal Comprehension; PRI, Perceptual Reasoning; WMI, Working Memory; and PSI, Processing Speed.

Bold values are significant at *p* < 0.05.

A total of 49 individuals (72.1% of the T1 sample) agreed to take part at T2. Their ages ranged from 24 to 74 years (Mean 48.4 years), including 25 younger (mean 36.2 years; five female) and 24 older adults (mean 60.9 years; five female; for details see Table 2).

**Table 2:** T2 age-group comparisons of autistic traits, mental health, and QoL.

Measure	Age groups (*N* = 49)		Statistics		
	Younger (*n* = 25) Mean (SD)	Older (*n* = 24) Mean (SD)	*t*(47)	*p*	*Cohen’s d*
Age	**36.23 (7.64)**	**60.94 (6.85)**	**−11.86**	**<0.001**	**−3.39**
FSIQ^a^	111.18 (18.86)	116.05 (17.35)	−0.81	0.43	−0.27
VCI^a^	113.06 (14.64)	116.47 (14.25)	−0.71	0.48	−0.24
PRI^a^	110.12 (19.41)	114.21 (16.82)	−0.68	0.50	−0.23
WMI^a^	109.00 (18.26)	116.44 (16.13)	−1.28	0.21	−0.43
PSI^a^	101.71 (21.52)	106.39 (12.57)	−0.79	0.43	−0.27
*Autistic traits*					
SRS-2 Total	71.36 (10.63)	68.71 (12.61)	0.75	0.46	0.23
SRS-2 SCI	70.05 (10.60)	69.00 (12.74)	0.29	0.77	0.09
SRS-2 RRB	69.14 (11.08)	68.24 (12.36)	0.25	0.80	0.08
*Mental health*					
Anxiety	15.65 (11.01)	11.46 (9.49)	1.09	0.28	0.12
Depression	17.24 (11.50)	14.50 (12.12)	0.88	0.39	0.23
Somatoform disorder	0.22 (0.43)	09 (0.30)	0.89	0.38	0.35
Major depressive syndrome	0.22 (0.43)	0.18 (0.40)	0.25	0.80	0.10
Other depressive syndrome	0.17 (0.38)	09 (0.30)	0.56	0.58	−0.97
Panic syndrome	0.17 (0.38)	0.18 (0.40)	−0.10	0.92	−0.05
Other anxiety syndrome	0.28 (0.46)	0.18 (0.40)	0.57	0.57	0.22
Bulimia Nervosa	0.06 (0.24)	-	0.78	0.44	0.33
Binge eating Disorder	0.11 (0.32)	0.09 (0.30)	0.74	0.87	0.06
Alcohol abuse	0.06 (0.24)	0.18 (0.40)	−1.07	0.30	−0.38
Extent of daily difficulties	1.00 (0.84)	0.73 (0.65)	0.92	0.37	0.36
*Quality of life*					
PWI outcome variables					
Subjective Well-being	**34.04 (12.88)**	**43.24 (10.43)**	**−2.59**	**0.013**	**−0.79**
Global Life Satisfaction	46.96 (17.69)	57.14 (24.73)	−1.58	0.12	−0.47
WHOQOL-BREF outcome variables					
Overall-QoL	**51.25 (17.16)**	**66.67 (21.41)**	**−2.54**	**0.015**	**−0.79**
Health-QoL	46.25 (26.00)	55.95 (29.48)	−1.12	0.27	−0.35
Support received	46.43 (26.73)	43.75 (30.96)	0.26	0.80	−0.09
Physical-QoL	58.90 (19.18)	62.29 (17.47)	−0.59	0.56	−0.18
Psychological-QoL	**48.45 (17.55)**	**62.29 (17.47)**	**−2.09**	**0.044**	**−0.65**
Social-QoL	**40.60 (18.84)**	**55.00 (16.99)**	**−2.57**	**0.014**	**−0.80**
Environmental-QoL	60.75 (18.31)	70.05 (11.29)	−1.97	0.056	−0.61

a FSIQ, Full-Scale IQ; Index Scores: VCI, Verbal Comprehension; PRI, Perceptual Reasoning; WMI, Working Memory; and PSI, Processing Speed.

Bold values are significant at *p* < 0.05.

Reasons for non-participation at T2 (*n* = 19, 28% of the T1 sample) were: chronic or terminal illness (*n* = 7), or death (*n* = 2); lost to follow-up, moved to different town, city or country (*n* = 2); work commitments, personal difficulties, or family commitments (*n* = 5); administrative reasons, or self-exclusion or withdrawal from the project for other reasons (*n* = 3).

#### Community involvement

Participant well-being was central to all stages of the research. During the study scoping and design, advice was sought from autistic peer researchers in co-creation discussion forums. Participants were consulted throughout the data collection about any adaptations they might require to the study procedures and materials, and every effort was made to incorporate adaptations to meet their individual needs. These adaptations included easy read formats for information about study aims and task instructions, changes to sensory stimuli in the research laboratory, e.g., noise reduction and soft lighting, and frequent breaks between tasks as needed. Every effort was made to ensure that these adaptations did not compromise the methodology or quality of the data.

### Procedure

Difficulties related to autistic traits, mental health, and QoL were compared at two time-points: Study 1 focused on T1 cross-sectional comparisons between younger and older groups; Study 2 followed the same participants who took part in T1 and applied the same procedures regarding ethics, sample selection, materials, and assessment. The mean follow-up interval was 2.4 years. At each time point, the measures were conducted face to face in a single session.

### Measures

Assessments of IQ, autistic traits, mental health (anxiety, depression), daily functional difficulties, and QoL were carried out at T1 (see Table 1) and repeated at T2 follow-up.

In addition to the measures described below, participants provided information on co-occurring physical and mental health conditions, difficulties related to everyday skills (e.g., self-care, household management, employment, and managing finances), social interaction difficulties, loneliness and isolation, sensory sensitivities, and stress responses experienced as meltdowns and/or shutdowns and related cognitive difficulties in everyday life. This information was captured through semi-structured questions, using the Passport to Individual Autism Support (PIAS), developed by the National Autistic Society (2012).

#### Intellectual ability profiles

The Wechsler Adult Intelligence Scales–Fourth Edition (WAIS-IV) was administered at T1 and T2 follow-up. The WAIS-IV is a widely used standardised measure to assess intellectual ability profiles in adults aged 16–90 years (Wechsler, 2008). It comprises 10 core and five supplemental subtests, providing Index scores for Verbal Comprehension (VCI), Perceptual Reasoning (PRI), Working Memory (WMI) and Processing Speed (PSI), and a composite Full-Scale IQ (FSIQ) score.

#### Autistic traits

At T1, clinical reports confirmed autism diagnosis (see the section Sample Characteristics). Since those reports incorporated a variety of measures, the Autism Diagnostic Observation Schedule–2nd Edition (ADOS-2; Lord et al., 2012) and the Social Responsiveness Scale–2nd Edition (SRS-2; Constantino and Gruber, 2012) were administered to confirm diagnostic reports. The Module 4 (adult) ADOS assessment is reported to have sensitivity of 0.61 (Bastiaansen et al., 2011; de Bildt et al., 2016); specificity is between 0.50 and 0.84 (Maddox et al., 2017). The first author was trained in ADOS-2 administration to 0.89 reliability and overseen by a certified ADOS trainer. The ADOS-2 was administered to 50 participants (74%) who consented to complete this assessment. Over a third (37.2%) of assessments in this study were double-coded for inter-rater reliability which was maintained at 0.84 or above. We note that subsequent development of calibrated severity scores (CSS; Hus and Lord, 2014) are now more commonly used as more sensitive measures of autism symptom severity in adults. However, because the CSS algorithm was not available when the present study data were collected, the ADOS data reported here are according to the algorithm in Lord et al. (2012).

The SRS-2 is a self-rated measure of autism-related traits and difficulties based on the DSM-5 (American Psychiatric Association, 2013) criteria for autism. It provides a Total score and separate domain index scores (T-scores; range 30 to >90, mean 50, SD 10) for Social Communication, Social Motivation, Restricted Interests and Repetitive Behaviours (RRBs), Social Awareness, and Social Cognitive functioning. Studies with autistic adults have reported sensitivity and specificity of 0.85 and 0.83, respectively (Bölte, 2012). The SRS-2 was administered at T1 and T2 explore possible age-related changes over time.

#### Physical and mental health

##### Passport to individual autism support

**Figure 1:**
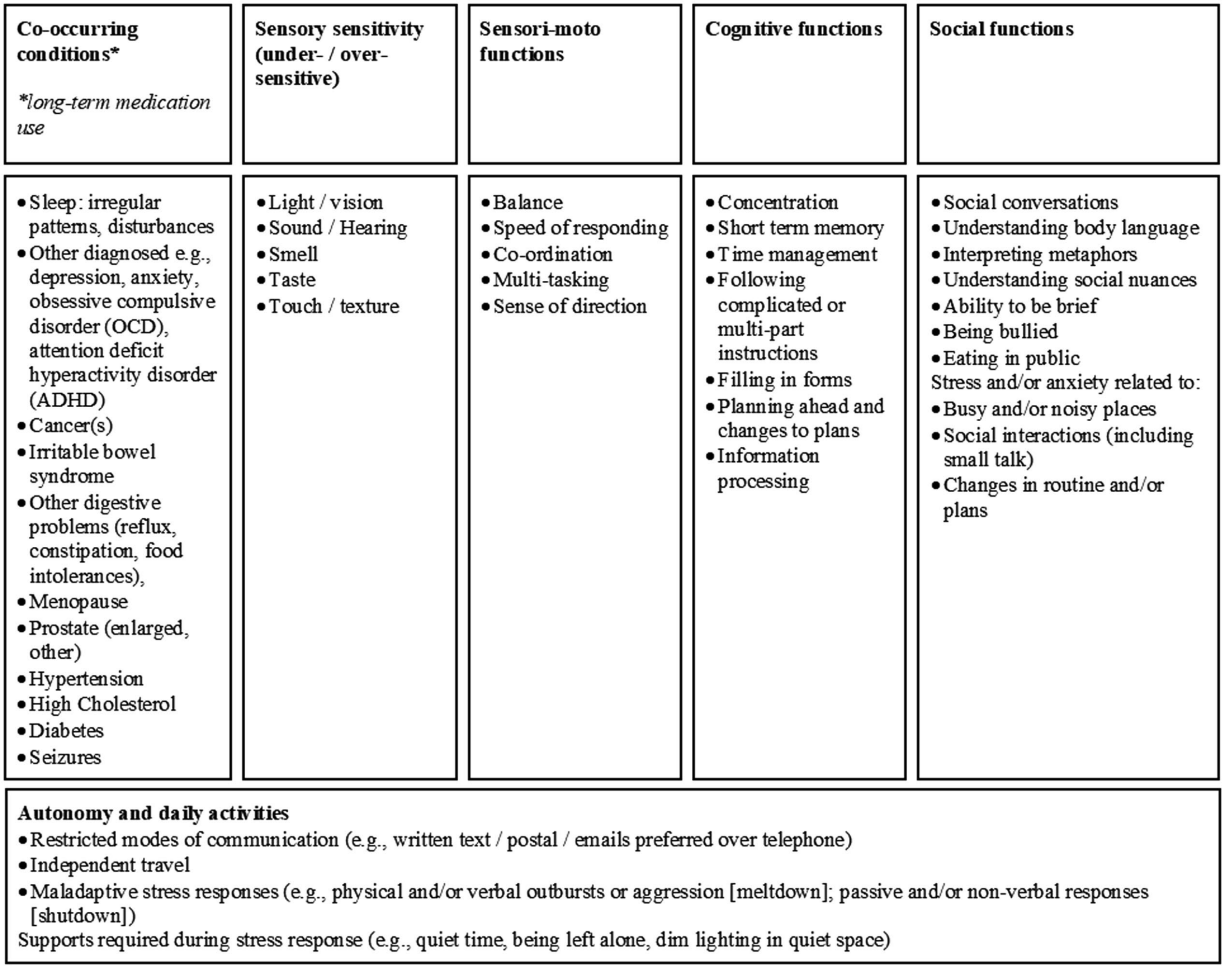
Self-reports of difficulties experienced by autistic adults, reported on the Passport to Individual Autism Support (PIAS, National Autistic Society, 2012).

The PIAS was designed by the National Autistic Society to assist autistic individuals who have difficulties advocating for themselves when accessing health and social care services. The resulting information (see Appendix 3 in Supplementary material; Figure 1) provides a summary of co-occurring conditions and other self-reported difficulties associated with autism, such as sensory sensitivities, limited motor function, and difficulties related to cognitive processing and social interactions (National Autistic Society, 2012).

##### Patient health questionnaire–9-item

The PHQ is a standardised assessment based on DSM-IV (American Psychiatric Association, 1994) diagnostic classifications for psychiatric conditions. The PHQ is a self-rated questionnaire comprising core items designed to screen for depression and other conditions, including anxiety and panic syndrome, somatoform symptoms (e.g., pain and digestive problems), risk of eating disorders and alcohol abuse. A single item reflects everyday functional difficulties: *“How difficult have these problems made it for you to do your work, take care of things at home, or get along with other people?”* Scores determine clinical diagnostic thresholds for anxiety- and depression-related conditions, indicated by a minimum number of symptoms, yielding accuracy of 0.85, sensitivity of 0.75, and specificity of 0.90 (Spitzer et al., 1999). Participants completed the PHQ at T1 and T2, based on symptoms experienced during the previous 4-week (major depression, panic/other anxiety syndromes); 2 weeks (other depressive syndrome); 3 months (eating disorders); or 6 months (alcohol abuse). Additionally, a single item question evaluated the degree of everyday difficulty experienced from any reported symptoms. Reliability in the present sample was excellent, with Cronbach’s *α* 0.93 (Spitzer et al., 1999).

##### Beck anxiety inventory–second edition

The BAI-II is a 21-item self-rated standardised measure that captures the physical symptoms associated with anxiety that cannot be explained by biological reasons (e.g., hypoglycaemia; peripheral neuropathy, or other non-anxiety factors). Item scores, rated on a scale from 0 (“not at all”) to 3 (“severely”), provide a Total anxiety score (0–63) and clinical cut-offs indicating the severity of anxiety symptoms, yielding 0.92 reliability, and 0.75 test–retest reliability (Beck and Steer, 1993). The BAI-II was administered at T1 and T2. In the present sample, reliability was good, with Cronbach’s *α* 0.88 (Beck and Steer, 1993).

##### Beck depression inventory–second edition

The BDI-II is a 21-item self-rated standardised measure, based on DSM-IV-TR (American Psychiatric Association, 1994) diagnostic criteria, that is widely used to screen for depression and related physical and psychological symptoms, e.g., suicidal ideation, rumination, sleep disturbances, weight loss, and change in appetite in adolescents and adults. Item scores, rated on a scale from 0 (“not at all”) to 3 (“severely”) provide a Total depression score (0–63), and clinical cut-offs indicating the severity of depression symptoms, yielding 0.86 reliability and 0.90 test–retest reliability (Beck et al., 1996). The BDI-II was administered at T1 and T2. In the present sample reliability was excellent, with Cronbach’s *α* 0.94 (Beck et al., 1996).

#### Quality of life and subjective well-being

At the time this study began, very few investigations had evaluated QoL in autism and even fewer had explored QoL in older autistic adults (e.g., Geurts and Vissers, 2012). Accordingly, two QoL measures were used (see below); these are designed to capture similar domains but use different methods of calculating outcome scores. Thus, a conversion formula (see International Well-being Group, 2013) was applied to the World Health Organisation Quality of Life–Short Form (WHOQOL-BREF) scores for Overall-QoL, Health-QoL, and degree of Support received, for comparable reporting in relation to the PWI-A scores. Both the WHOQOL-BREF and the PWI-A were administered at T1 and T2 to evaluate any (positive or negative) change in quality of life and well-being.

##### World health organisation quality of life–short form

The WHOQOL-BREF assesses the effects of physical and cognitive difficulties on everyday living and QoL. Items are self-rated on a Likert-type scale from 1 (“worst”) to 5 (“best”). The measure provides a Total score and four domains outcome scores (all 0–100; mean 50), namely: Physical-QoL (e.g., activities of daily living, sleep, pain, and illness), Psychological-QoL (e.g., negative/positive feelings and memory/concentration), Social-QoL (e.g., relationships and social support), and Environmental-QoL (e.g., financial status, living arrangements, and access to and quality of social care). Three additional questions provide measures of Overall-QoL, Health-QoL, and degree of Support received from others. One of the benefits of the WHOQOL-BREF is that it asks about the individual’s satisfaction with life-domains rather than being based on normative assumptions about what constitutes a “good” quality of life (e.g., having a range of friends). In the present sample, reliability was good, with Cronbach’s *α* 0.85 (Skevington et al., 2004).

##### Personal well-being index, adult

The PWI is a self-rated standardised measure of quality of life that focuses on *subjective* well-being (SWB) and global life satisfaction (GLS). It has good index reliability (Cronbach alpha 0.70–0.85; International Well-being Group, 2013), and 0.84 test–retest reliability (Cummins and Law, 2005; Lau and Cummins, 2005). Seven core items evaluate “health,” “standard of living,” “relationships,” “safety,” “achievement,” “future prospects,” and “community,” with scores averaged to provide a measure of SWB. Two optional questions evaluate GLS (item-1: “Thinking about your own life and personal circumstances, how satisfied are you with your life as a whole?”) and Religion (item-8: “How satisfied are you with your spirituality or religion?”). Each item is rated from 0 to 10 (0 = “no satisfaction at all,” 10 = “completely satisfied”). Because almost half the participants (42%, *n* = 29) did not answer the optional item about religion, data for this item were excluded from the overall analysis. In the present sample, reliability was good, with Cronbach’s *α* 0.88 (International Well-being Group, 2013).

### Statistical analysis

T1 and T2 cross-sectional comparisons were carried out with *t*-tests between younger and older groups. Statistical significance (alpha, *p* < 0.05) and effect sizes (*d*) are reported for between-group contrasts. A secondary analysis was carried out using Bonferroni corrections for multiple comparisons (*p* < 0.001). At the time of this study, there was no precedence for evaluating analysis of change in autistic adults. The general ageing literature was consulted to inform the analytic approaches to the data reported here. In our study, analysis of change (Table 3) was calculated using individual change scores for each participant, followed by a SD method of variance between T1 and T2 scores for each participant to establish a reliable change index (RCI; see Jacobson and Truax, 1991; Frerichs and Tuokko, 2005), using the formula: X2-X1/SD, where X2 represents the individual score at T2 (averaged for each Age Group) and X1 represents the individual score at T1 (averaged for each Age Group), and SD is the T1 standard deviation of the mean for each Age Group. The RCI scores +1 indicate change; scores >+1SD indicate improved change, whereas scores <−1 SD indicate deterioration (Frerichs and Tuokko, 2005, p. 324). A detailed description of the method is provided in Supplementary material (Appendix 4). Additionally, to check the above calculation outcomes, paired *t*-tests were run to confirm any group differences between T1 and T2 scores (see Table 3).

**Table 3:** Change scores between T1 and T2.

Measure	Follow-up sample (*n* = 49)					Statistics	
	T1 Mean (SD)	T2 Mean (SD)	CI 95%	RCI†	*t*(47)	*p*	*Cohen’s d*
*Autistic traits* *^a^*							
SRS-Total	70.01 (11.58)	70.08 (11.81)	66.51–73.96	−0.04	1.50	0.14	−0.01
SRS-SCI	69.53 (11.56)	70.85 (12.90)	65.74–75.96	−1.33	0.00	1.00	−0.11
SRS-RRB	68.70 (11.59)	72.52 (12.85)	65.13–77.60	0.13	**2.22**	**0.03**	**−0.31**
*Mental health* *^b^*							
Anxiety	13.40 (8.92)	13.83 (10.42)	9.94–17.73	−0.34	0.26	0.80	−0.04
Depression	16.39 (13.35)	16.00 (11.66)	11.72–20.45	−0.34	0.18	0.85	0.03
*Quality of life* *^c^*							
PWI outcome variables							
Support	45.00 (27.39)	48.28 (26.67)	34.77–58.42	0.04	−0.21	0.83	−0.12
SWB	38.43 (12.53)	38.90 (13.41)	33.79–44.00	−1.02	−1.07	0.29	−0.04
GLS	51.82 (21.70)	54.14 (19.91)	45.22–61.71	−0.49	−0.57	0.57	−0.11
WHOQOL-BREF outcome							
Overall	59.15 (20.73)	60.00 (20.34)	52–40–67.60	−0.62	−1.07	0.29	−0.04
Health	51.22 (27.92)	52.50 (24.87)	42.41–61.79	−0.38	−0.57	0.57	−0.05
Physical	60.63 (18.17)	58.33 (16.63)	52.12–66.37	−0.83	1.47	0.15	0.13
Psychological	54.49 (18.83)	52.17 (16.62)	41.94–58.37	−0.73	0.17	0.84	0.13
Social	47.98 (19.13)	44.40 (19.45)	37.14–54.01	−0.68	−0.20	0.84	0.19
Environmental	65.51 (15.66)	63.83 (16.04)	57.84–70.45	−1.00	0.31	0.76	0.11

† RCI is the Reliable Change Index score calculated as the difference between T1 and T2 scores.

a Autistic traits (SRS-2): Total and index (*T*-scores) for Social Communication (SCI) and Restricted Interests and Repetitive Behaviours (RRBs) are reported (range 30 to ≥90), where higher scores indicate greater related difficulties.

b Mental health total scores reported for Anxiety (BAI-II, range 0–63), and Depression (BDI-II, range 0–63), where higher scores indicates greater related difficulties.

c Quality of life scores (PWI and WHOQOL-BREF, range 0–100) are reported for Subjective well-being (SWB), Global life satisfaction (GLS), Support received from others, and quality of life (QoL) domains related to Overall-QoL, Health-QoL, Physical-QoL, Psychological-QoL, Social-QoL, and Environmental-QoL, where higher scores indicate better quality of life or support.

Cohen’s d effect sizes indicate small (*d* = 0.20–0.49) effect of change scores. See Supplementary material for detailed description of change analysis (Appendix 4).

At T1 and T2, multivariate linear regression analyses were carried out to determine Overall-QoL and Health-QoL as outcome variables, using a stepwise regression method for autistic traits and mental and physical health conditions as independent variables. Stepwise backward regression analysis was used to assess which factors were predictors of Health and Overall QoL outcomes. This method was used since the data were exploratory and no prior theoretical basis for selecting particular variables as predictors over other variables. Furthermore, the stepwise backward method controls for suppression effects in analysing the relative contribution of each variable to the regression model. The following variables were significant predictors and were subsequently included in a second regression using the Enter method: age, processing speed, self-report autistic traits, self-report RRBs, anxiety, depression, Somatic complaints, and difficulties in daily living. At the time this study was conducted, this statistical approach was a recommended method (e.g., Howell, 2002) for the exploratory investigation in this study. Alternative approaches have subsequently been suggested (e.g., Smith, 2018), but these statistical guidelines were not available at the time the present study was conducted.

### Missing data analysis

In the typical ageing literature, longitudinal studies of this nature report participant attrition between 10 and 32% depending on the age and gender of participants (Young et al., 2006), the duration of the study and intervals between follow-up assessments (Saiepour et al., 2019). In the present study, every effort was made to collect completed data sets for all participants. However, where background information or test data were not available (e.g., ADOS), these are reported as missing data [Little’s MCAR test: χ^2^ (632) = 555.06, *p* = 0.99; see Appendix 2 in Supplementary material].

## Study 1: T1 cross-sectional

### Results of T1 study

#### Sample characteristics

Table 1 describes the sample characteristics at T1. Given the study design, younger (*n* = 37) and older (*n* = 31) groups differed significantly on chronological age. Groups did not differ on years of formal education, despite five older adults holding fewer. There were also no group differences in gender [*χ*^2^(1) = 0.005, *p* = 0.94], or any IQ scale scores (see Table 1).

Table 4 summarises autistic profile scores for younger and older adults. There were no age group differences in ADOS-2 or SRS-2 scores (Table 4). As previously mentioned, all participants had existing clinical diagnoses of an autism spectrum condition. Although 12 participants (younger, *n* = 7; older, *n* = 5) did not meet the cut-off for ADOS-2 Total scores for ‘Autism Spectrum’ (≥7), they did meet or exceeded the cut-off for both index scores (Communication ≥2; Social Interaction ≥4). These findings are consistent with variable sensitivity and specificity of the ADOS for adults who also have co-occurring mental health conditions (Maddox et al., 2017).

**Table 4:** T1 comparisons of autistic traits in younger and older adults.

Measure	Age Group		Statistics		
	Younger (*n* = 25) Mean (SD)	Older (*n* = 25) Mean (SD)	*t*(48)	*p*	*Cohen’s d*
*Autistic traits*					
ADOS-2 Total^a^	8.44 (2.83)	9.76 (3.83)	−1.39	0.17	−0.39
ADOS-2 Comm.^a^	2.88 (1.48)	3.48 (1.33)	−1.51	0.14	−0.43
ADOS-2 Social Int.^a^	5.56 (2.16)	6.28 (2.77)	−1.01	0.32	−0.29
ADOS-2 RRB^a^	1.41 (1.30)	0.95 (0.95)	1.33	0.19	−0.17
	Younger (*n* = 33) Mean (SD)	Older (*n* = 27) Mean (SD)	*t*(58)	*p*	*Cohen’s d*
SRS-2 Total^b^	72.61 (9.60)	69.00 (12.79)	1.25	0.22	0.32
SRS-2 SCI^b^	71.36 (9.68)	69.41 (12.48)	0.68	0.50	0.17
SRS-2 RRB^b^	71.39 (10.27)	68.96 (12.48)	0.83	0.41	0.21

a ADOS-2 indices (threshold scores): Total (≥7); Comm, Communication (≥2); Social Int, Social Interaction (≥4); and RRB, Restricted interests and repetitive behaviours (≥1).

b SRS-2 T-index scores (threshold ≥ 57): Total; SCI, Social Communication Index. RRB, Restricted Interests and Repetitive Behaviours.

#### Physical and mental health

**Figure 2:**
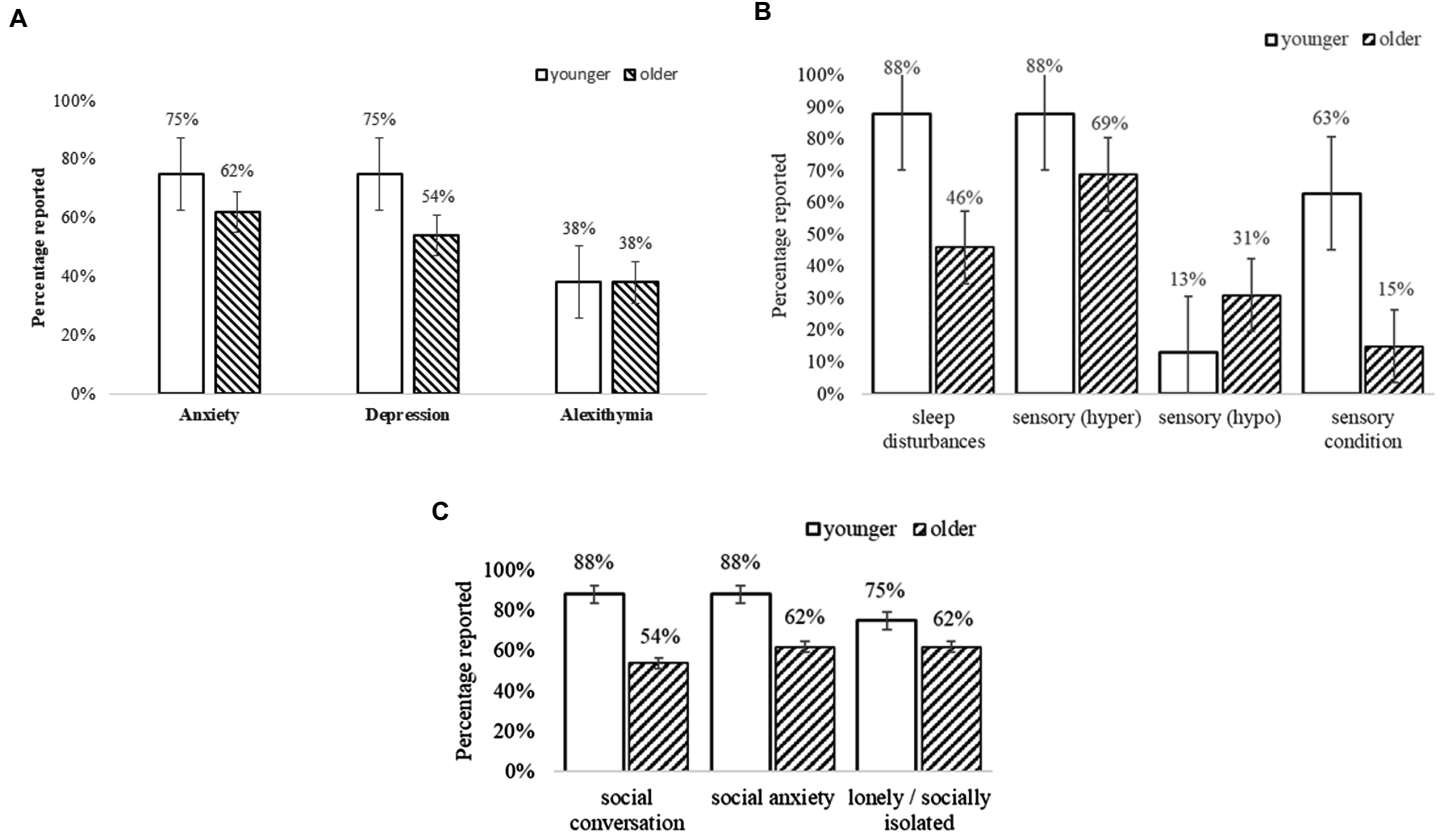
**(A)** Self-reported mental health conditions. Error bars indicate 1 SE. **(B)** Self-reported sensory and sleep conditions. Error bars indicate 1 SE. **(C)** Self-reported social difficulties. Error bars indicate 1 SE.

Figure 2 summarises the PIAS self-reported data related to everyday difficulties with social skills, mental and physical health indicated high rate of co-occurring conditions in younger and older adults. Overall, participants reported high rates of symptoms related to anxiety (*n* = 50 [74%]) and depression (*n* = 47 [69%]), and related difficulties in identifying and describing emotions (alexithymia, *n* = 28 [41%]; for further information see Appendix 3 in Supplementary material; Figure 1). Sleep disturbances (e.g., difficulty falling asleep; frequent waking) were common in more than half to two-thirds of adults (*n* = 48 [71%]) as were sensory hypersensitivities (*n* = 58 [85%]). Conditions related to sensory sensitivities (e.g., visual, auditory, touch, taste/texture, and olfactory, *n* = 27 [40%]) were reported more by younger than older adults, whilst only five adults (younger, *n* = 1; older, *n* = 4) reported hyposensitivity or sensory-seeking. Social difficulties and stresses were related to social conversation (*n* = 26 [39%]), social anxiety (*n* = 28 [42%]), and loneliness or social isolation (*n* = 26 [39%]). Additionally, somatic conditions that presented greatest difficulties were related to neurophysiological symptoms (heart racing, *n* = 10 [14%]; shortness of breath, *n* = 14 [21%]); digestive problems (bowel, *n* = 14 [21%]; indigestion, *n* = 28 [41%]); and pain (stomach, *n* = 19 [28%]; back, *n* = 10 [14%]; and joints or limbs, *n* = 28 [41%]).

**Figure 3:**
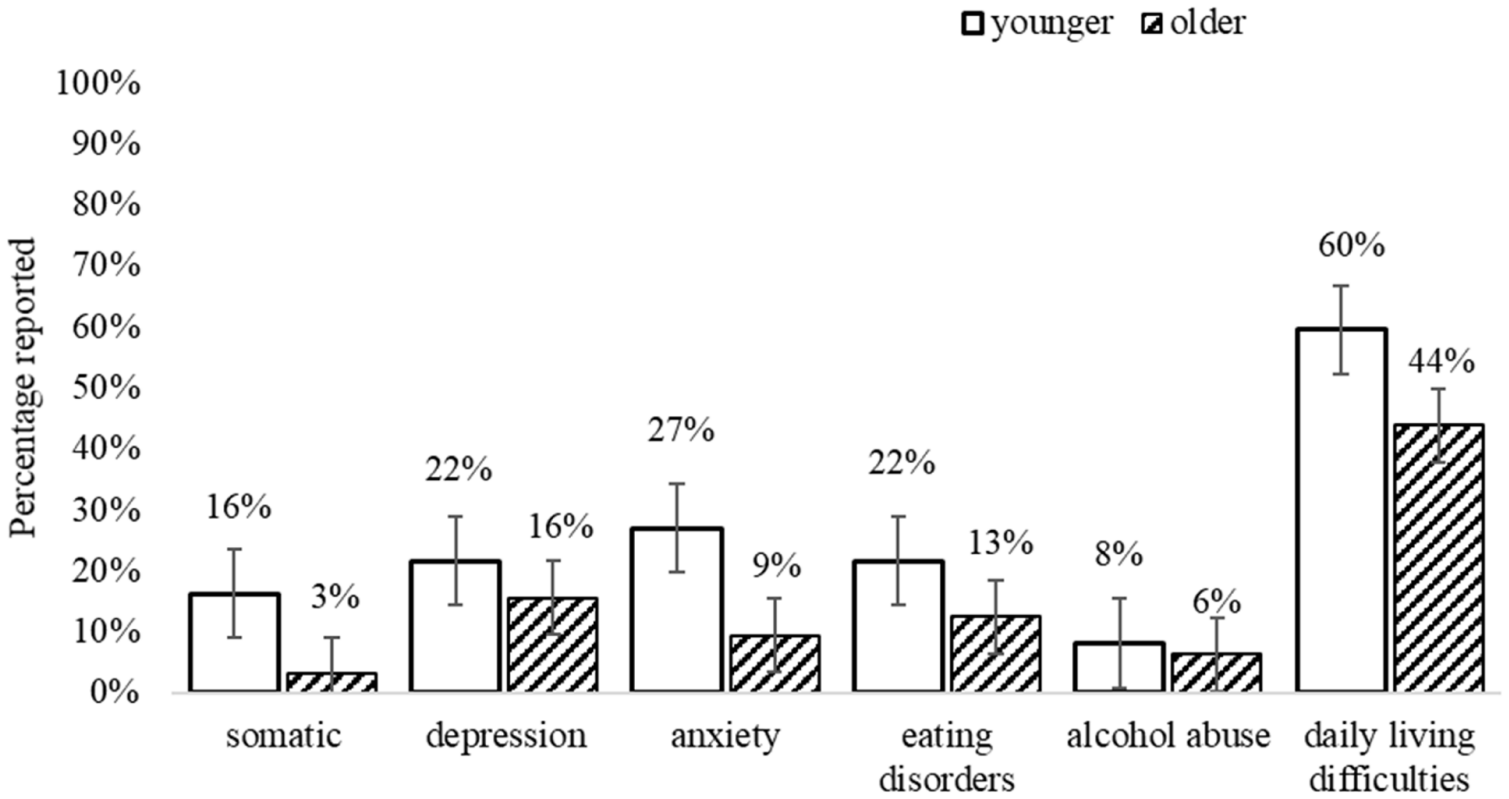
Co-existing conditions and everyday difficulties (measured by PHQ). Error bars indicate 1 SE.

Figure 3 summarises the percentage of adults who met the threshold for at least one co-occurring condition (measured by PHQ) and experienced everyday difficulties related to those conditions. Overall, at T1 59% of the younger (*n* = 22) and 30% of older adults (*n* = 9) met the criteria for at least one other condition. The number of co-occurring mental health conditions ranged from 0 to 4, with almost half (46%) of all autistic adults having multiple co-occurring conditions. Although there were no significant differences between younger and older adults on any mental health measures (Table 5), 37% of younger (*n* = 14) and 22% of older adults (*n* = 7) met the criteria for three or more co-occurring mental health conditions. Both groups reported being on multiple pharmacological treatments for those conditions, which aligns with the self-report background data collected (using the PIAS; see Appendix 3 in Supplementary material).

**Table 5:** T1 comparisons of mental health and QoL in younger and older adults.

Measure	Age group		Statistics		
	Younger (*n* = 29) Mean (SD)	Older (*n* = 25) Mean (SD)	*t*(52)	*p*	*Cohen’s d*
*Mental health*					
Anxiety^a^	16.28 (9.12)	12.24 (8.93)	1.63	0.11	0.45
Depression^b^	19.11 (12.54)	14.08 (13.76)	1.41	0.17	0.38
Somatoform disorder^c^	0.22 (0.42)	0.04 (0.21)	1.84	0.07	0.54
Major depressive syndrome^c^	0.30 (0.47)	0.17 (0.39)	1.00	0.32	0.45
Other depressive syndromes^c^	0.00 (0.00)	0.04 (0.21)	−1.09	0.28	−0.30
Panic syndrome^c^	0.15 (0.36)	0.04 (0.21)	1.22	0.23	0.35
Other anxiety syndrome^c^	0.22 (0.42)	0.09 (0.29)	1.30	0.20	0.37
Bulimia nervosa^c^	0.04 (0.19)	0.00 (0.00)	0.92	0.36	0.27
Binge Eating disorder^c^	0.26 (0.45)	0.17 (0.39)	0.72	0.48	0.20
Alcohol abuse^c^	0.11 (0.32)	0.09 (0.29)	0.28	0.78	0.08
Extent of daily difficulties^c^	**1.44 (0.83)**	**1.01 (0.83)**	**2.33**	**0.024**	**0.67**
*Quality of life*					
PWI outcome variables					
Subjective Well-being^d^	**35.10 (12.36)**	**42.08 (11.72)**	**−2.11**	**0.04**	**−0.58**
Global life satisfaction^d^	50.67 (20.50)	57.08 (24.93)	−1.04	0.30	−0.28
WHOQOL-BREF outcome variables					
Overall-QoL^e^	**52.38 (17.51)**	**65.22 (22.28)**	**−2.11**	**0.04**	**−0.64**
Health-QoL^e^	46.43 (25.35)	57.61 (29.61)	1.34	0.19	−0.41
Support^e^	46.43 (23.73)	43.75 (30.96)	0.26	0.80	0.10
Physical-QoL^e^	57.35 (18.93)	62.30 (17.56)	−1.19	0.24	−0.34
Psychological-QoL^e^	**47.85 (17.67)**	**60.25 (18.27)**	**−2.40**	**0.02**	−0.68
Social-QoL^e^	47.46 (19.49)	53.33 (17.01)	−1.71	0.09	−0.32
Environmental-QoL^e^	62.15 (17.97)	69.08 (14.44)	−1.50	0.14	−0.43

a Anxiety (BAI-II) calculated for Total score (range 0–63); clinical cut-off scores: 0–7, “Minimal”; 8–15, “Mild”; 16–25, “Moderate”; and 26–63, “Severe.”

b Depression (BDI-II) calculated for Total score (range 0–63); clinical cut-off scores: 1–10, “normal ups-and-downs”; 11–16, “Mild mood disturbance”; 17–20, “Borderline clinical depression”; 21–30, “Moderate depression”; 31–40, “Severe depression”; and >40, “Extreme depression.”

c Clinically significant mental health conditions (PHQ). Daily difficulties: 0 = “not difficult at all”; 1 = “somewhat difficult”; 2 = “very difficult”; 3 = “extremely difficult.”

d Subjective well-being and Global life satisfaction (PWI). Scores range from 0 to 100 (mean = 50, SD = 15).

e Quality of Life domains and Support received from others (WHOQOL-BREF). Scores range from 0–100 (mean = 50, SD = 15).

Bold values are significant at *p* < 0.05.

The most common conditions reported by younger adults were Anxiety (27%; of which 16.2% other Anxiety syndromes; 10.8% Panic syndrome), Major Depressive syndrome (21.6%), Eating disorders (21.6%; of which 18.9% Binge Eating; 2.7% Bulimia Nervosa), Somatic disorders (16.2%, e.g., bodily pain), and Alcohol abuse (8.1%). For older adults, the most common conditions were Depression (15.6%; of which 12.5% Major Depressive syndrome; 3.1% other Depression syndrome), Binge Eating disorder (12.5%), Anxiety (9.4%; of which 6.3% other Anxiety syndromes; 3.1% Panic syndrome); Alcohol abuse (6.3%), and Somatic disorders (3.1%). Moreover, both groups reported difficulties in everyday functioning (e.g., doing housework, employment, and social relationships), as “somewhat” to “very difficult,” related to these conditions.

Table 5 summarises the statistical analyses for T1 comparisons between younger and older adults, on the standardised assessments of mental health and quality of life. Although the standardised measures captured a lower rate of mental health concerns than those self-reported in background descriptive information (using the PIAS), these were nevertheless still predominant for the majority of adults, indicating at least “moderate anxiety” symptoms (as measured by BAI-II), and “mild mood disorder” to “borderline clinical depression” symptoms (as measured by BDI-II), which were also observed on the PHQ (reported in Table 5).

#### Quality of life and subjective well-being

As set out in Table 5, scores across SWB and QoL domains were, overall, poor for both younger and older adults, indicated by below-average scores (<50; scale 0–100) on the PWI and WHOQOL-BREF scales, respectively (see Roestorf and Bowler, 2016; Roestorf, 2018 for reports of significant lower QoL for autistic adults compared to non-autistic groups). Moreover, both groups reported low degree of Support received for their everyday needs. The most common indicators of low SWB were related to lack of Personal Relationships and feeling isolated from the Community; lack of Achievement; and concerns about Health and Future. Scores for these factors were also below normative population mean scores of 70–80 points (see Cummins et al., 2003; International Well-being Group, 2013). Standard of Living and feeling safe (Safety) were amongst the highest SWB indicators.

#### Predictors of quality of life at T1

Table 6 sets out the main predictors of QoL domains across all participants. Age was not consistently related to QoL [all *r*(44) < 0.17, all *p* > 0.25] as low QoL scores, across domains, were observed across the lifespan. The only exceptions to this were Overall QoL and Social QoL domains, for which older adults reported greater satisfaction. Overall, depression and anxiety symptoms (as measured by BDI-II and PHQ, and the BAI-II, respectively) were the strongest consistent predictors of Global Life Satisfaction and Subjective Well-being, and most QoL domains including Overall-QoL, Health-QoL, Physical-QoL, Psychological-QoL, and Environmental-QoL. However, these symptoms did not predict Social-QoL scores [*R^2^* = 0.07; *F*(2,46) = 1.83, *p* > 0.05].

**Table 6:** T1 predictors of quality of life.

Measure		*B*	*SE*	Beta	*t(52)*	*p*
PWI outcome variables						
*Subjective well–being*						
Depression		−0.53	0.11	−0.60	**−4.70**	**0.003**
Anxiety		−11.05	4.69	−0.30	**−2.36**	**0.025**
Social Responsiveness Scale – Total		−0.54	0.12	−0.53	**−4.33**	**<0.001**
*Global Life Satisfaction*						
Depression		−1.22	0.17	−7.21	**−7.27**	**<0.001**
Social Responsiveness Scale – Total		−0.56	0.26	−0.30	**−2.19**	**0.033**
WHOQOL-BREF outcome variables						
*Overall-QoL*						
Depression		−0.65	−26	−0.44	**−2.55**	**0.017**
Social Responsiveness – Total		−0.56	0.25	−0.33	**−2.22**	**0.032**
*Health-QoL*						
	Depression	−0.75	0.34	−0.36	**−2.22**	**0.03**

Bold values are significant at *p* < 0.05.

Difficulties related to autistic traits (as measured by SRS-2 Total scores), predicted Subjective Well-Being, and to a much lesser extent Overall QoL, Global Life Satisfaction, Psychological-QoL, and Environmental-QoL.

By contrast, factors related to age, gender, and autistic traits (as measured by ADOS-2 and SRS-2 Communication and RRB scores) did not predict any QoL or SWB outcomes [all *F*(8,30) < 1.60, all *p* > 0.05].

### Discussion of T1 results

At T1, there were no age group differences in autistic traits (Table 4) or mental health (Table 5). Although older adults reported slightly better Social-QoL and Overall-QoL, in general QoL was low in both groups. Poor QoL was strongly linked to depression symptoms, anxiety, and autistic features, and was associated with difficulties in everyday functioning (e.g., autonomy, self-care, doing housework, holding employment, and maintaining social relationships; and *cf.* Park et al., 2019). The high rates of co-occurring physical and mental health conditions identified in the present study concur with many recent reports of everyday difficulties and poorer QoL in younger and older autistic adults (e.g., Khanna et al., 2014; Bishop-Fitzpatrick et al., 2016; Mason et al., 2018; Lawson et al., 2020). The findings replicate observations of recent studies that outline comparisons between older autistic and non-autistic adults (see Yarar Zivrali et al. (in press); and see Van Heijst and Geurts, 2015; Ayres et al., 2017 for reviews).

## Study 2: T2 longitudinal follow-up

### Results of T2 study

#### Sample characteristics

Table 2 summarises the characteristics T2 participants (25 younger and 24 older adults). The mean interval between T1 and T2 assessments was 2.4 years (range 1.2–3.8 years) and was not significantly different between age groups [*t*(25) < 1.00, *p* > 0.05]. Comparisons of the profiles of participants who continued to the T2 follow-up with those who did not, revealed significantly greater T1 depression symptoms in the non-continuing group [*t*(36) = 2.33, *p* = 0.03, *d* = 0.78], but no other differences in T1-derived cognitive or health profiles.

Autistic traits profiles (as measured by the SRS-2; Constantino and Gruber, 2012) were re-assessed in younger and older participants. As at T1, there were no age-related group differences at T2.

#### Mental health

As indicated in Table 2, there were no significant Age Group differences in anxiety or depression symptoms (measured by the BAI-II and BDI-II, respectively). Nor were there any differences in PHQ-measured symptoms of somatic complaints (e.g., bodily pain), or eating disorders, or alcohol abuse. Once again, daily difficulties related to co-occurring conditions were rated by both groups as “somewhat” to “very difficult.”

#### Quality of life and subjective well-being

Table 2 summarises the T2 QoL scores. As at T1, QoL at T2 was low across domains. The T2 data followed a similar pattern to T1 observations, with older adults once again reporting greater satisfaction in Subjective Well-being, Overall-QoL, and Social-QoL. However, in contrast to T1 data, older adults also reported greater Psychological-QoL than younger adults.

#### Analysis of change over time

Table 3 indicates the change in T1-T2 scores that were assessed by comparing individual scores for autistic traits, mental health (anxiety, depression), and QoL, following the procedure set out earlier (see section “Statistical analysis”). There were no significant differences between T1 and T2 scores on any mental health or QoL domain scores [all *t*(26) < 1.96, all *p* > 0.05]. Regarding autistic traits, only RRBs showed significant change presented as increased at T2 [*t*(26) = 2.22, *p* < 0.04]. However, the analysis was not significant after applying Bonferroni corrections (*p* < 0.001) for multiple analysis (*p* = 0.16, *η*_p_^2^ = 0.13).

#### Predictors of quality of life at T2

Table 7 summarises the main predictors of T2 QoL for all autistic adults. Once again, Age did not predict any QoL outcomes, across domains [all *r*(29) ≤ 0.17, all *p* ≥ 0.18], which followed the pattern observed at T1. However, Age at T2 was significantly correlated with Subjective well-being [*r*(29) = 0.40, *p* = 0.015] and Global Life Satisfaction [all *r*(29) = 0.34, *p* = 0.036], which was explained by higher satisfaction in these domains reported by older autistic adults (see Table 2).

**Table 7:** T2 Predictors of quality of life.

Measure	B	*SE*	Beta	*t*(47)	*p*
PWI outcome variables					
*Subjective Well-being*					
Depression	−0.38	0.18	−0.38	**−2.12**	**0.044**
Anxiety	−0.44	0.20	−0.39	**−2.22**	**0.036**
Autistic traits RRBs	−0.97	0.31	−0.88	**−3.18**	**0.005**
Difficulty in daily living	−6.55	2.90	−0.44	**−2.26**	**0.035**
*Global life satisfaction*					
Depression	−0.58	0.24	−0.39	**−2.40**	**0.027**
Autistic traits RRBs	2.00	0.98	1.14	**2.05**	**0.05**
WHOQOL-BREF outcome variables					
*Overall-QoL*					
Depression	−0.04	0.01	−0.53	**−3.22**	**0.004**
*Health-QoL*					
Anxiety	−0.04	0.02	−0.39	**−2.55**	**0.018**
Depression	−0.04	0.01	−0.49	**−3.24**	**0.004**
Eating disorder	1.58	0.71	0.31	**2.24**	**0.036**
*Physical-QoL*					
Anxiety	−0.85	0.25	−0.50	**−3.43**	**0.002**
Depression	−0.59	0.22	−0.40	**−2.74**	**0.012**
Autistic traits RRBs	0.63	0.30	0.46	**2.09**	**0.05**
Eating disorder	34.31	11.44	0.39	**3.00**	**0.007**
*Psychological-QoL*					
Anxiety	−0.59	0.25	−0.36	**−2.36**	**0.027**
Depression	−0.73	0.24	−0.51	**−3.08**	**0.005**
Difficulty in daily living	−12.43	3.69	−0.52	**−3.37**	**0.003**
*Social-QoL*					
Anxiety	−0.84	0.34	−0.46	**−2.47**	**0.023**
Autistic traits Social Communication	−14.68	4.52	−0.55	**−3.25**	**0.004**
Difficulty in daily living	4.81	2.00	2.84	**2.41**	**0.026**
*Environmental-QoL*					
Anxiety	−0.57	0.23	−0.43	**−2.53**	**0.021**
Depression	−11.80	5.03	−0.32	**−2.35**	**0.030**
Autistic traits RRBs	−0.49	0.23	−0.38	**−2.15**	**0.040**
Eating disorder	−20.01	5.60	−0.49	**−3.58**	**0.002**

Bold values are significant at *p* < 0.05.

Autistic traits were significantly negatively correlated with all QoL domains, except for Health-QoL, (all *r*(29) ≥ 0.32, all *p* < 0.05). Given the marginal increase in autistic traits observed at T2, these were also explored in relation to QoL outcomes. RRBs, but not social communication or total scores (measured by SRS-2), were a significant predictor of Subjective Well-being (*R^2^* = 0.14, *F*(1,27) = 4.43, *p* < 0.05), Physical-QoL (*R^2^* = 0.2, *F*(1,27) = 4.35, *p* = 0.05), and Environmental-QoL (*R^2^* = 0.14, *F*(1,28) = 4.62, *p* < 0.05).

Difficulties related to anxiety, depression, and eating disorders were consistent predictors of Health-QoL [*R^2^* = 0.60, *F*(3,22) = 11.16, *p* < 0.001], Physical-QoL [*R^2^* = 0.65, *F*(3,22) = 13.34, *p* < 0.001; Table 7]. Whilst depression alone predicted Overall-QoL [*R^2^* = 0.51, *F*(2,23) = 12.16, *p* < 0.001], and anxiety and difficulty in everyday living predicted Psychological-QoL [*R^2^* = 0.50, *F*(2,23) = 11.42, *p* < 0.001]. Social-QoL, however was predicted by multiple factors including anxiety, depression, and autistic traits difficulties related to social communication, and difficulty with everyday living [*R^2^* = 0.71, *F*(6,19) = 7.80, *p* = 0.005]. The significant predictors of respective QoL outcomes are presented in Table 7.

## Discussion of T2 results

There were no overall changes in autistic traits over time (as measured by SRS-2). In relation to quality of life outcomes, although older autistic adults reported significantly elevated satisfaction, compared to younger adults, in domains of Subjective Well-being, Overall-QoL, Psychological-QoL, and Social-QoL, statistical comparisons between T1 and T2 outcomes showed no general improvement in QoL, over time. Similarly, there were no changes in mental health, again reflecting continuing difficulties in this group, and poor QoL across domains (*cf.* also Mason et al., 2018; Lai et al., 2019). Furthermore, co-occurring depression symptoms at T2 were a consistent significant predictor of all QoL outcomes. Overall, these findings mirror the pattern of associations observed at T1 (Table 6).

## General discussion

In the present study, we set out to describe the patterns of autism traits and well-being in the context of ageing, by exploring age-related differences between younger and older autistic adults. We also explored changes that occurred over a short follow-up (approximately 2 years) period. This paper describes our findings related to general ability, autistic traits, mental and physical health, and several quality of life domains. The present findings concur with emerging literature that highlights how increased difficulties related to autistic traits and mental health (e.g., anxiety, depression; Lawson et al., 2020; Oakley et al., 2021; Mason et al., 2022) are strong predictors of poor quality of life in autistic adults. The results highlight specific areas of concern for autistic adults, as well as domains that may contribute toward a more positive QoL in older age.

Overall, existing data from cross-sectional and longitudinal studies suggest that the core features of autism remain relatively stable over time (e.g., Magiati et al., 2014; Gotham et al., 2015; Lever and Geurts, 2016; Steinhausen et al., 2016), including up to middle age (Howlin et al., 2013). However, low well-being and poor QoL outcomes are frequently reported for autistic people, particularly in adulthood (e.g., Ayres et al., 2017; Mason et al., 2018; Lai et al., 2019; Lawson et al., 2020). Poor mental health is also known to have adverse effects on cognitive abilities, social isolation, and QoL (e.g., McClintock et al., 2010; and see Lai et al., 2019; Mason et al., 2019), whereas increased facilitation of social integration is linked to higher QoL and fewer anxiety and depression symptoms (Lever and Geurts, 2016; McConachie et al., 2018; Mason et al., 2018) but this is not well understood in older autistic adults (Mason et al., 2019). Therefore, accounting for individual differences is an important consideration for future autism ageing studies.

The pattern of findings in the present study broadly reflects the findings in previous studies. In our study, no age-related changes were observed for most outcome measures. Regarding autistic traits, social communication difficulties remained generally stable, although there was some increase in restricted interests and repetitive behaviours (RRBs) from T1 to T2.

Restricted Interests and Repetitive Behaviours were also a significant predictor of QoL outcomes related to Subjective Well-being, Global Life Satisfaction, and Environmental-QoL. The underlying causes of these associations are unknown but it may be that continuing difficulties associated with RRBs could adversely impact environmental autonomy, related to the home environment, access to and quality of health and social care, and participation in community activities or opportunities for leisure and recreation (e.g., Oakley et al., 2021; and see Park et al., 2019).

Similarly, age-neutral outcomes were observed across mental health and QoL domains, over time. Thus, whilst there were no further significant declines, overall, in these domains, nor were there any improvements. At T1, around two-thirds of participants reported co-occurring physical and mental health conditions which were associated with poorer QoL. Anxiety and depression were experienced by more than two-thirds of the autistic adults in the study and difficulties related to both of these conditions were significant predictors of poor QoL at T1 and T2. Mental health difficulties were also strongly associated with everyday difficulties (e.g., housework, employment, social relationships; and *cf.* Gotham et al., 2015; Mason et al., 2018; Park et al., 2019). Accordingly, the findings at both time points suggest that anxiety and depression have a widespread impact on many aspects of everyday life, including participation in social activities (see Park et al., 2019 for similar findings in and adolescent-young adult sample). In the present study, reliability across the mental health and quality of life measures used was good (>0.85) to excellent (0.94). These findings concur with previous reports of sustained difficulties related to mental health and QoL (see Roestorf and Bowler, 2016; Roestorf, 2018; Yarar Zivrali et al., in press, for cross-sectional comparisons with non-autistic groups; and see, e.g., Gotham et al., 2015; Van Heijst and Geurts, 2015; Lever and Geurts, 2016; McConachie et al., 2018).

Given that these difficulties still remained significant at T2, the findings raise important issues about the mental health and well-being needs of autistic adults in the context of ageing. However, the direction of any association is unknown and the underlying causes and exacerbating factors related to these difficulties need to be systematically explored in future research (see Lord et al., 2021 for current review and future-focused recommendations).

### Study limitations and future directions

The main limitation of the present study relates to the generalisability of the findings to the wider autistic community. In common with most other research that directly includes autistic people (i.e., not *via* proxy reports), the data are based on relatively small volunteer groups of participants with average to above-average cognitive skills. The ADOS is not designed to account for age-related differences or trajectories over time and revised ADOS CSS algorithm (Hus and Lord, 2014) were not available at the time. There is emerging literature to suggest the revised algorithm can provide a more robust evaluation of differences in symptom profiles and behavioural outcomes. However, there is still little evidence to support its use and sensitivity in the context of ageing and autism (Morrier et al., 2017). Moreover, we do not know if the same associations would be found in participants with more severe autistic or psychiatric conditions, or by those less able to share their own experiences and difficulties, or engage social participation without individual supports (e.g., Charlton et al., 2022). Similarly, whether the pattern of results reported here would be mirrored in a more intellectually disabled sample, particularly in low-middle income countries where resources for post-diagnostic support and health care are more scarce is an open question (e.g., McCauley et al., 2020; Frankish and Horton, 2021).

The findings are also limited by a lack of detailed information on variables such as socioeconomic status. Whilst we did record years of education, measures of income, employment status, and residence were not systematically collected, and this further compromises the generalisability of these findings to autistic adults living in different circumstances. The inclusion of non-standardised measure of physical health status was primarily due to the lack of an autism-specific measure in this area, but again is a methodological concern that should be considered when interpreting these findings. Although the measures selected to assess mental health and well-being were based on the best available at the time, that had also been used in previous autism research, more autism-specific measures have since been developed, such as for assessing anxiety (e.g., Rodgers et al., 2020) and Quality of Life (e.g., ASQOL; McConachie et al., 2018 but see Williams and Gotham, 2021 for caution on interpreting the ASQoL composite score). The present study did not report the internal consistency of the standardised general population measures that were used with an autistic community sample. Therefore, caution should be exercised in interpreting the present study findings, subject to future replication. However, we note that more recent literature has validated the use of measures, such as the BDI-II (Williams et al., 2021) and PHQ (Arnold et al., 2020) with good construct validity and reliability, respectively, in autistic adult samples.

In the present study, only around two-thirds of participants at T1 went on to complete the T2 follow-up. This was primarily because of participant ill-health or death, life commitments, or withdrawal from the larger research programme. It is possible that the demands of ongoing research participation may have been too challenging for some adults with greater cognitive, functional or mental health difficulties, or that poorer health, or lower Socio-Economic Status may have affected the ability or means to take part in the follow-up study (e.g., Howlin et al., 2014; Van Heijst and Geurts, 2015).

A third limitation centres on gender. Like the majority of other autism studies, most of our participants were male. Although some recent studies on gender differences suggest that masking of autism-related symptoms by autistic females may underpin more pronounced mental health difficulties (e.g., Mandy, 2019). Fombonne (2020) highlights the poor methodological quality of much research in this area (and see Williams et al., 2021). Therefore, better representation of autistic women in the context of ageing research is needed.

A fourth limitation is the statistical analyses used in the present study. Given the present study is one of the few longitudinal studies of autistic adults, there was little precedent for the exploratory investigation of change in this study. Furthermore, there is still little consensus in the general ageing literature on the “right” reliability of change analysis, since change scores are influenced by the type of assessment, cognitive and mental “health” of participants at baseline compared to follow up, and the duration the of interval between test and retest, and the heterogeneity of the participant group (Ivnik et al., 2000; Maassen, 2001; Frerichs and Tuokko, 2005). To evaluate the predictors of quality of life, stepwise regression was used based on a review of the literature available at the time of this study. Whilst alternative approaches have subsequently been suggested (e.g., Smith, 2018), these guidelines were not available at the time the present study was completed. Moreover, while the present study did not demonstrate significant age effects over time, the validation of “age-neutral” outcomes is required from replication studies. The findings should therefore be interpreted with caution and subject to replication in future studies using alternative statistical analyses.

A final limitation is the short time between the T1 and T2 assessments, which may have reduced the chances of detecting significant patterns of change. This coupled with the problems of attrition noted above, points to the need for greater attention to be paid to reducing attrition rates especially in the context of longer follow-up studies.

### Strengths and contributions of the present study

The present findings provide new and important insights into health and well-being outcomes for autistic adults as they grow older. The majority of existing autism research relies on cross-sectional studies between autistic and non-autistic comparison groups (Raz et al., 2005). However, it is only in longitudinal evaluations that true *changes* over time can be observed (Salthouse, 2004).

In the present study, both cross-sectional and longitudinal methods were used to assess age related changes across a wide range of standardised and self-report measures of autistic traits, health and well-being. This comprehensive approach enabled us to identify and evaluate the factors that are associated with ageing and autism to a better understanding of well-being outcomes for autistic adults as they grow older.

### Conclusion

To our knowledge, the present study is among the first to combine cross-sectional and longitudinal methodologies, across a breadth of measures, to assess mental health and quality of life in a community-based sample of younger and older autistic adults. The findings highlight the adverse effects of co-occurring physical and mental health conditions on everyday living and quality of life over time. Thus, the present research contributes to furthering our understanding of the specific challenges that may be associated with ageing and autism. However, more work is needed on larger, more representative cohorts, with sustained longitudinal follow-ups at multiple time points. Only through continued efforts can we understand the potential factors that may help or hinder transitions across the lifespan (Roestorf and Lambrechts, pre-print; https://osf.io/ygkw5/) and support autistic individuals to lead longer, healthier, and happier lives.

## Data availability statement

The datasets presented in this article are not readily available because the raw data supporting the conclusions of this article are governed by General Data Protection Regulations (2008) in the EU and UK. Accordingly, no data, whether anonymised or identifiable, may be made shared without the express written consent of participants involved in this research. Requests to access the datasets should be directed to amanda.roestorf.2@city.ac.uk.

## Ethics statement

The studies involving human participants were reviewed and approved by City, University of London Psychology Department Research Ethics Committee PSYETH(UPTD) 13/14 28. The patients/participants provided their written informed consent to participate in this study.

## Author contributions

DB and PH were involved in obtaining research funding and providing supervision to AR during the programme of work, and reviewed and advised on the research protocol, analysis, and results and contributed to editing the manuscript. AR designed all studies and conducted all data collection, diagnostic assessment, and analysis and writing of the manuscript. All authors contributed to the article and approved the submitted version.

## Funding

This research was supported by funding from the Medical Research Council UK in collaboration with the National Autistic Society for a 4-year CASE Industry Studentship (grant no. MR/K016911/10).

## Conflict of interest

The authors declare that the research was conducted in the absence of any commercial or financial relationships that could be construed as a potential conflict of interest.

## Publisher’s note

All claims expressed in this article are solely those of the authors and do not necessarily represent those of their affiliated organizations, or those of the publisher, the editors and the reviewers. Any product that may be evaluated in this article, or claim that may be made by its manufacturer, is not guaranteed or endorsed by the publisher.

## Footnotes

^1^The term autism is used in this manuscript to reflect the general profile of autistic features related to Autism spectrum disorder. We acknowledge that while there is still ongoing discussion in the autistic and scientific communities about the preferred terminology when referring to diagnosed individuals, the present paper has used ‘identity-first’ language (i.e., autistic individual; autistic adult) as this was identified as the preferred terminology for autistic people involved in our study (and see Kenny et al., 2016; Bottema-Beutel et al., 2021).

## Supplementary material

The Supplementary materials for this article can be found online at: https://www.frontiersin.org/articles/10.3389/fpsyg.2022.741213/full#supplementary-material
