# Supplementary material for: Ageing and autism: A longitudinal follow-up study of mental health and quality of life in autistic adults
Source: Front Psychol. 2022 Aug 23;13:741213. doi: 10.3389/fpsyg.2022.741213 (PMC9447441; doi:10.3389/fpsyg.2022.741213)
Supplement: Supplementary file 1 [file Data_Sheet_1.docx]

# Supplementary Material

# Appendix 1. Measures of IQ, Language and Communication

## General ability

At Time 1, all participants completed assessments of general intellectual ability as measured by the Wechsler Adult Intelligence Scales–Fourth Edition (WAIS-IV; Wechsler, 2008), as part of the larger programme of work (see Roestorf, 2018). The WAIS-IV is a widely used standardised measure of IQ, with normative scores (mean 100, standard deviation 15). Raw scores from the 10 sub-tests are computed as age-stratified standardised scores, resulting in Index scores for Verbal Comprehension (VCI), Perceptual Reasoning (PRI), Working Memory (WMI) and Processing Speed (PSI), and the composite FSIQ score.

### Language and communication

All participants used spoken language as their primary method of communication. In addition to the verbal comprehension index measured by the WAIS-IV, participants also completed the Comprehensive Receptive and Expressive Vocabulary Test–Third Edition (CREVT-3; Wallace & Hammill, 2013). Those data are reported in (Roestorf, 2018), but mentioned here for clarification of the overall methods used to establish English language proficiency in the present study sample.

# Appendix 2. Missing Data Analysis

Before analysing the data, a Missing Value Analysis (MVA) was carried out using an Expectation Maximisation (EM) method in SPSS (V. 24). The MVA confirmed that any missing data at T2 were Missing Completely at Random (MCAR), according to Little’s (1995) MCAR test. Overall, the MVA EM analysis confirmed no significance of missing data patterns between Age Groups, (Little's MCAR test: χ^2^ (632) = 555.06, *p* = .99).

Analysis of the profile differences between T1 and T2 samples highlighted several factors related to attrition. In the younger adults, 69% of the group variance was explained by autism-related communication difficulties ($\beta$ = .89, *t* = 4.09, *p* < .005) and depression ($\beta$ = .57, *t* = 2.62, *p* < .04). For the older adults, autism-related communication difficulties alone accounted for 67% of the sample variance ($\beta$ = .45, *t* = -2.38, *p* < .05) and anxiety which accounted for a further 30% ($\beta$ = -.57, *t* = -2.97, *p* < .009).

The missing data are reported in the Tables within the main manuscript and discussed in interpreting the study findings.

# Appendix 3. Passport to Individual Autism Support (National Autistic Society, 2012)

The PIAS is available from the National Autistic Society’s website ([www.autism.org.uk](http://www.autism.org.uk)) for completion by the individual or their carer or advocate. An adapted version of the PIAS was administered as a semi-structured interview to capture functional difficulties, and background diagnostic and medical history (see Supplementary Materials Appendix 3, Figure 1 for a description of domains captured by the PIAS).

[insert Figure 1 about here]

Moreover, the following physical health conditions were reported as pre-existing diagnosed conditions for which participants were either currently or previously prescribed treatment (asterisks * indicate medications taken):

Anxiety*; Depression*; Alexithymia; Dyslexia; Dyspraxia; Stammering; Selective mutism; Attention Deficit Hyperactivity Disorder*; Bipolar disorder*; Obsessive-Compulsive Disorder; Schizophrenia or Schizoid Personality Disorder; other mental health conditions (e.g. behavioural or eating disorders); Parkinson’s disease or early-stage symptoms*; Respiratory conditions (e.g. asthma; chronic obstructive pulmonary disease); Physical or mobility-limiting conditions (e.g. arthritis; fibromyalgia; osteoporosis)*; Cancer (e.g. bowel; colon; ovarian)*; Heart disease*; high Cholesterol*; Hypertension*; Diabetes*; Sensory conditions (e.g. Psoriasis; Rhinitis)*; Migraine; Food intolerances; Digestive or Bowel conditions (e.g. Irritable Bowel syndrome)*; Absent Seizures; Hormonal or Endocrine conditions (e.g. prostate; menopause; thyroid)*; Enuresis and/or Encopresis.

The main manuscript discusses these self-report data in the context of how co-existing conditions affect autistic adults’ experience of everyday difficulties.

# Appendix 4. Methods used for analysis of change using Reliable Change Index method

The longitudinal analysis included two methods of analysis to detect statistical change from T1 to T2 scores (see Frerichs & Tuokko, 2005, for a review of the methods summarised below). The first assessment of change followed the Standard Deviation (SD) method, using the formula X2-X1/SD where X2 represents the individual score at T2 (averaged for each Diagnostic Group) and X1 represents the individual score at T1 (averaged for each Diagnostic Group), and SD is the T1 standard deviation of the mean for each Diagnostic Group. The calculation results in SD-change scores, where +1 SD indicates change. Respectively, scores > +1SD indicate improved change, whereas scores < -1 SD indicate deterioration.

A secondary analysis used the Reliable Change Index (RCI) method to “correct for measurement error and practice effects” (Frerichs & Tuokko, 2005, p. 324). RCIs are the statistical methods used for “determining the significance of test score changes in serial neuropsychological assessment of older adults” (Frerichs & Tuokko, 2005, p. 321). The RCI method has been widely used to assess change scores between two or more time-points (i.e. T1 to T2) as a measure of cognitive change in older typically ageing adults (Woods et al., 2006; Frerichs & Tuokko, 2005; Gavett, Ashendorf & Gurnani, 2015). This approach has been also applied to behavioural assessment of autistic children (see Barber, 2012), in assessing longitudinal outcomes on a given measure. The RCI is calculated as (X2-X1) - (M2-M1)/SED, where X2 is the individual participants’ scores at T2 and X1 is their score at T1; M2 is the mean score for each Diagnostic Group at T2, and M1 is the group mean score at T1. The Standard Error of the difference score (SED) is calculated as the Standard Deviation of the mean observed difference (Mdiff) score. In order to calculate these scores, M1 and M2 are calculated first, obtaining Mdiff and SED accordingly. The RCI was then applied to individual difference scores (X2-X1), using the above formula. The confidence interval (CI) for these scores were calculated for 95% (SE x 1.96) and 90% (SE x 1.645), as recommended by Frerichs & Tuokko (2005). Difference scores that are outside the CI range (above or below) are indicators of reliable change. For cognitive assessments and QoL measures, scores that fall below the CI indicate decline or ageing-negative outcomes, and those scores above CI indicated improvement or ageing-positive outcomes, whilst scores that fall within the CI are deemed stable or ageing-neutral outcomes. Accordingly, better cognitive and wellbeing outcomes refer to ageing-neutral or ageing-positive RCIs. Whereas, for the clinically relevant assessments, such as the degree of autistic traits as measured by the SRS-2 and measures of anxiety and depression, a decline in symptoms or ageing-negative RCI would be suggestive of better outcomes.

In order to confirm the results arising from the above methods, paired sample t-tests were used to confirm test-retest (T1 to T2) change scores. The appropriateness of each statistical method relative is addressed in the larger programme of work (Roestorf, 2018) in relation to a reliable and meaningful evaluation of change in ageing and ASD.
